# Subfunctionalization of duplicated genes as a transition state to neofunctionalization

**DOI:** 10.1186/1471-2148-5-28

**Published:** 2005-04-14

**Authors:** Shruti Rastogi, David A Liberles

**Affiliations:** 1 Computational Biology Unit, BCCS, University of Bergen, 5020 Bergen, Norway

## Abstract

**Background:**

Gene duplication has been suggested to be an important process in the generation of evolutionary novelty. Neofunctionalization, as an adaptive process where one copy mutates into a function that was not present in the pre-duplication gene, is one mechanism that can lead to the retention of both copies. More recently, subfunctionalization, as a neutral process where the two copies partition the ancestral function, has been proposed as an alternative mechanism driving duplicate gene retention in organisms with small effective population sizes. The relative importance of these two processes is unclear.

**Results:**

A set of lattice model genes that fold and bind to two peptide ligands with overlapping binding pockets, but not a third ligand present in the cell was designed. Each gene was duplicated in a model haploid species with a small constant population size and no recombination. One set of models allowed subfunctionalization of binding events following duplication, while another set did not allow subfunctionalization. Modeling under such conditions suggests that subfunctionalization plays an important role, but as a transition state to neofunctionalization rather than as a terminal fate of duplicated genes. There is no apparent selective pressure to maintain redundancy.

**Conclusion:**

Subfunctionalization results in an increase in the preservation of duplicated gene copies, including those that are neofunctionalized, but never represents a substantial fraction of duplicate gene copies at any evolutionary time point and ultimately leads to neofunctionalization of those preserved copies. This conclusion also may reflect changes in gene function after duplication with time in real genomes.

## Background

A number of mechanisms can generate duplicate copies of genes, ranging from single gene duplications to regional and whole genome duplications [1-3]. Large increases in gene number have been coupled to increases in organismal complexity and radiative divergence at several points in the history of metazoans including during the chordate/vertebrate transition and during the teleost fish divergence [1,4,5].

Metazoans differ from prokaryotes in their much smaller effective population sizes, where theory predicts that neutral stochastic processes will be relatively more important than adaptive processes in the expected case that adaptive mutations are rarer than nearly neutral mutations [6]. Large scale analyses, based upon the ratio of nonsynonymous to synonymous nucleotide substitution rates [7] or MacDonald-Kreitman statistics [8] have indicated small to intermediate degrees of positive selection (adaptive substitutions) in mammals, but these clearly do not represent the majority of substitutions. In such studies, it appears to be specific positions in protein-encoding genes, rather than the genes as a whole that are under positive selection [7]. Even examining substitution as a neutral walk through sequence in a folded protein (ignoring positive selection) has shown such a process to have fairly complex dynamics [9]. From this, it is relevant to examine population genomic phenomena, like the fates of duplicated genes, in the context of physical models of proteins. Further, it is not possible to systematically identify fates of real genes (subfunctionalization to the exclusion of neofunctionalization or vice-versa), so modeling under increasingly realistic conditions is likely to be the best way to understand evolutionary mechanisms.

**Figure 1:**
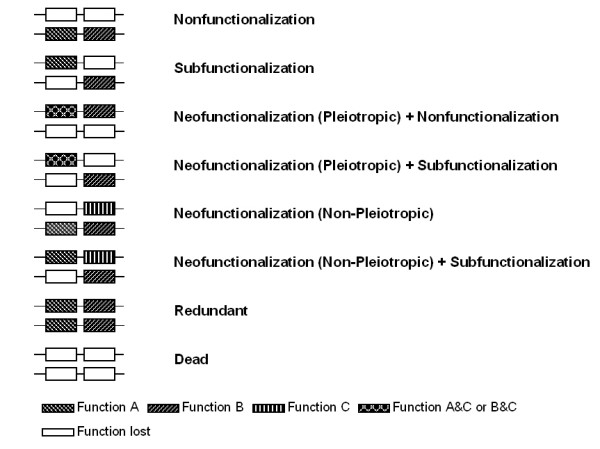
Eight different fates are possible in the two simulations, nonfunctionalization (pseduogenization) without cellular death, subfunctionalization, pleiotropic neofunctionalization plus either nonfunctionalization of the other copy or subfunctionalization, non-pleiotropic neofunctionalization, non-pleiotropic neofunctionalization also involving subfunctionalization, redundancy, and cellular death. Some of the fates have additional combinations of activity that are not represented in this figure. It should also be noted that some of the characterizations overlap. For example, pleiotropic neofunctionalization occurs in combination with nonfunctionalization, subfunctionalization, or is redundant.

Pseudogenization or nonfunctionalization is a purely neutral process that ultimately eliminates one of the duplicated copies as a functional gene and is the most common fate. Subfunctionalization, is an alternative neutral process that leads to an increase in organismal gene number for genes or functions that show modularity (one representative type of modularity is modeled here, but other types are also possible). Neofunctionalization is an alternative process leading to an increase in organismal gene number, but dependent upon rarer adaptive mutations. Neofunctionalization can include the evolution of a completely new binding capability (as modeled here) or modification/improvement of existing binding capabilities under positive selection after removal of pleiotropic constraint. These alternative fates are presented in the context of a lattice model in Figure 1.

Lattices are models of folded proteins in square or cubic shapes (a cubic lattice was employed here). The folding of a lattice is dictated by the contacts from amino acids that are not adjacent in the primary sequence (these contacts are present in the folded and unfolded states). Because lattices are small and the folding rules are simple, they can be used for evolving populations of proteins to study their structural properties.

Lattice models have previously been used to make important predictions about the behavior of proteins in evolutionary contexts, including their metastability [10] and the evolvability of new folds [11]. Lattices that bind to peptides [12] and small hydrophobic molecules [13] have been described and the latter used to show that subfunctionalization can lead to an increase in duplicate gene retention rates. Here, model genes that fold into lattices and bind peptides were duplicated, with neofunctionalization and subfunctionalization (simulation A) or just neofunctionalization (simulation B) as possible events that would preserve duplicated copies in a genome, with nonfunctionalization (pseudogenization) as an alternative fate (see Figure 1 and Table 1). The relative levels of duplicate gene preservation and the importance of both neofunctionalization and subfunctionalization were assessed.

**Table 1:** The initial amino acid sequences including binding sites and folding energies (RT units) are shown for the 10 proteins. Additionally, the three ligands for each protein are shown with their initial binding energies (also RT units) at the two sites (A in italics and B in bold). It should be noted that the two binding sites are adjacent in three dimensions and overlap by one amino acid.

	Sequences	Conf. Eng.	Binding Site A	Ligand A	Ligand Eng. at A	Binding Site B	Ligand B	Ligand Eng. at B	Ligand C	Ligand Eng. at A	Ligand Eng. at B
1	MS*K*TAQ*K*RLLKELQQLIKDSPPGIVAGPKSEN NIF*I*WDCLIQGPPDTPY**A**DG**V**FNAKLEF**P**KDY	-0.78	R**PAV**	YRGM	-0.84	R*KIK*	YSMD	-1.42	YLEG	+0.59	+0.40
2	MS*TP*ARRRLMRDFKRMKEDAPPGVSASP**L**PDN VMVWNAM**I**IGP**A**DTPYEDGTF*R*LLLEFDEEYP	-0.73	E**ALI**	EIIL	-1.07	E*RTP*	EERQ	-1.54	EKEP	+0.79	+0.58
3	MTTSKERHSVSKRLQQELRT**LL**MSGDPGITAF *PD*GD*N*LFKWVATLDGPKDTVYE**S**LKYKLTLEF	-0.68	T**LLS**	SILL	-0.51	T*NDP*	SDEW	-0.81	STYL	+0.61	+0.23
4	M*NM*SGIALSRLAQERKA**W**RKDHPFGFVAVP**T**K NPD*G*TMN**L**MNWECAIPGKKGTPWEGGLFKLRM	-0.88	D**LTW**	GFHW	-0.83	D*GMN*	GTFE	-1.22	GEEC	+0.29	+0.60
5	MSTPARKRLMRDFKRLQQDPPAGISGAPQD**N**N IMLW*NAV*IFGPDDTPWDG**G**TFKL**S**LQFSEDYP	-0.66	D**NSG**	GTDD	-0.86	D*NAV*	GTWF	-0.64	GIFH	+0.80	+0.53
6	MI*V*PYNLPLPGGVVPRMLITILGT**VKP**NANRI ALDF*Q*RGND*V*AFHFNPRFNENNRRVIVCNTKL	-1.00	R**VKP**	YLEE	-1.61	R*QVV*	YKII	-1.13	YQRD	+0.78	+0.92
7	M*T*EENSKSEALLDIPMLEQYLELV*G*PK*L*ITD**G** LAVFEKMMPGYVSVL**ES**NLTAQDKKGIVEEGH	-0.96	I**GSE**	ATEY	-0.84	I*LGT*	AIPG	-1.00	AEFF	+0.59	+0.90
8	MEAVIKVISSAC**K**TYCGKTSP**S**KKEIGA**M**L*S*L LQKEGLLMSPSDLYSPGSWDPITAAL*SQ*RAMI	-0.64	Q**MSK**	YWQY	-1.22	Q*SQS*	YEPG	-1.04	YRFF	+0.84	+0.75
9	MD*E*PP*A*DGALKRAEE**LK**TQ**A**N*D*YFKAKDYENA IKFYSQAIELNPSNAIYYGNRSLAYLRTECYG	-0.37	Y**AKL**	EITF	-0.71	Y*DED*	EKKT	-1.92	EMVR	+0.94	+1.32
10	MK*SR*RWFHPNITGV**E**AENLLLTRGVDGSFLA**R** **P**SKSNPGDLTLSVRRNGAVTHIKIQNTGDY*Y*D	-1.45	T**ERP**	ASER	-1.47	T*YRS*	AMDD	-1.25	ALIL	+1.18	+0.82

## Results and discussion

A set of 10 stably folded lattices was designed to each bind to 2 different ligands at overlapping sites. A third ligand was present in the cell, but did not bind at either site at the start of the simulation. The lattice was duplicated in a constant population of 1000 cells, where those cells that bound the third ligand were 5% more likely to appear in the next generation (a selection coefficient of 5% is arbitrary, but only serves as a scaler of the results). In each generation, 10% of molecules became nonfunctional at random through transcriptional knock-out. The fitness function required molecules to fold and genomes to have binding capabilities for the first two ligands. Cells were selected under the constraint that the first and second ligands needed to be bound, but could be bound by either molecule (subfunctionalization possible) in simulation A. The second simulation (simulation B) tightened this constraint and required both ligands to be bound to the same molecule (subfunctionalization not possible). In simulation B, only neofunctionalization is possible as a mechanism to preserve both copies non-redundantly. Neofunctionalization can occur through two mechanisms, a pleiotropic mechanism where the third ligand binds at a site that is also capable of binding one of the other two ligands and a non-pleiotropic mechanism, where the third ligand binds to an inactive site. The average values of each fate (from 10 different lattices) in each of the two simulations are shown in Tables 2, 3.

**Table 2:** For simulation A the final average (over 10 different peptides each repeated 10 times) frequency of each fate across generations is reported. Error bars are reported as the standard error of the mean.

A							
**Generations**	**Nonfunc.**	**Sub**	**Neo-P**	**Neo-NP**	**Neo-P+Sub**	**Neo-NP+Sub**	**Redundant**

1	40.27 ± 1.10	0.01 ± 0.01	8.71 ± 0.90	0.23 ± 0.08	0.00 ± 0.00	0.00 ± 0.00	949.11 ± 1.37
20	360.89 ± 5.67	1.80 ± 0.24	127.72 ± 0.59	10.22 ± 0.88	0.37 ± 0.10	0.11 ± 0.04	469.49 ± 9.90
40	421.28 ± 6.83	4.41 ± 0.50	217.26 ± 13.95	18.54 ± 1.49	3.63 ± 0.75	1.55 ± 0.31	294.25 ± 10.85
60	410.64 ± 7.58	5.95 ± 0.53	294.73 ± 14.91	25.71 ± 1.99	7.57 ± 1.10	2.86 ± 0.48	209.70 ± 9.83
80	381.06 ± 7.72	7.47 ± 0.59	356.11 ± 14.76	29.03 ± 2.09	14.37 ± 1.67	5.21 ± 0.75	161.78 ± 9.00
100	352.85 ± 8.02	8.02 ± 0.61	402.22 ± 14.33	30.66 ± 2.33	24.05 ± 2.37	7.46 ± 0.95	128.69 ± 7.76
120	329.56 ± 7.83	9.32 ± 0.79	432.93 ± 14.01	32.30 ± 2.73	31.85 ± 2.95	8.14 ± 1.08	109.06 ± 6.94
140	312.37 ± 6.97	10.15 ± 0.93	450.22 ± 13.10	30.22 ± 2.69	43.83 ± 3.24	12.14 ± 1.49	93.30 ± 5.98
160	291.73 ± 7.02	10.33 ± 0.83	473.25 ± 12.97	29.06 ± 2.69	49.20 ± 3.77	13.92 ± 1.72	83.90 ± 5.23
180	284.48 ± 6.47	11.58 ± 0.84	478.46 ± 12.60	27.93 ± 2.51	57.99 ± 4.42	14.78 ± 2.03	76.08 ± 4.81
200	274.61 ± 6.18	12.51 ± 1.00	481.93 ± 12.78	27.62 ± 2.68	68.17 ± 4.70	16.09 ± 2.27	71.22 ± 4.35

**Table 3:** For simulation B the final average (over 10 different peptides each repeated 10 times) frequency of each fate across generations is reported. Error bars are reported as the standard error of the mean.

**Generations**	**Nonfunc.**	**Neo-P**	**Neo-NP**	**Redundant**
1	60.78 ± 2.06	8.85 ± 0.88	0.17 ± 0.06	928.63 ± 2.03
20	495.42 ± 9.68	128.62 ± 10.05	10.41 ± 0.91	336.46 ± 8.71
40	550.78 ± 11.61	222.18 ± 14.30	18.90 ± 1.49	168.86 ± 7.35
60	525.49 ± 12.97	303.64 ± 15.17	25.79 ± 1.86	102.54 ± 5.14
80	486.51 ± 12.83	369.06 ± 14.85	29.59 ± 2.21	69.92 ± 3.64
100	451.13 ± 12.54	418.99 ± 14.29	30.86 ± 2.37	52.86 ± 2.55
120	420.75 ± 12.05	457.10 ± 13.89	31.39 ± 2.45	44.07 ± 1.90
140	397.89 ± 11.73	488.39 ± 13.43	28.02 ± 2.25	38.97 ± 1.69
160	382.95 ± 11.24	509.07 ± 12.82	26.47 ± 2.24	34.40 ± 1.54
180	377.63 ± 10.28	518.26 ± 11.70	24.83 ± 2.24	31.15 ± 1.50
200	370.23 ± 9.59	529.04 ± 10.87	22.63 ± 2.10	29.79 ± 1.21

**Figure 2:**
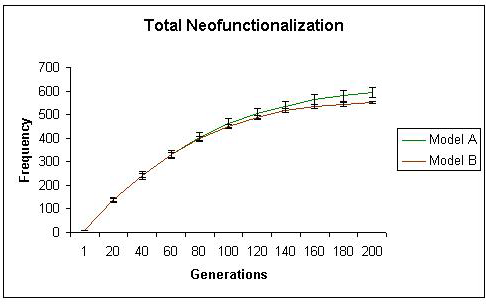
The total frequency of cells with neofunctionalized molecules (capable of utilizing ligand C) is shown for simulation A in green and simulation B in red. With time, the total level of neofunctionalization in simulation A surpasses that of simulation B.

While initially, both models generate similar levels of neofunctionalization, with time model A begins to show significantly more neofunctionalization. In model A, the total number of subfunctionalized genes, including those that have also neofunctionalized increased initially, but then reached a plateau. These results are shown in Figures 2 (neofunctionalization), 3 (subfunctionalization), and 4 (nonfunctionalization, including those that have also neofunctionalized on the other copy). It is clear that allowing subfunctionalization results in a greater retention rate of duplicate genes with less nonfunctionalization, although subfunctionalization without neofunctionalization never accounts for a large fraction of the duplicate genes at any point in evolutionary time (total terminal preservation of both duplicates is shown in Figure 5). Figure 5 indicates that the retention profile is completely different when subfunctionalization occurs compared to when it does not. It is also clear in these simulations that there is not a strong selective pressure to retain robustness through redundancy, as seen in Figure 6.

**Figure 3:**
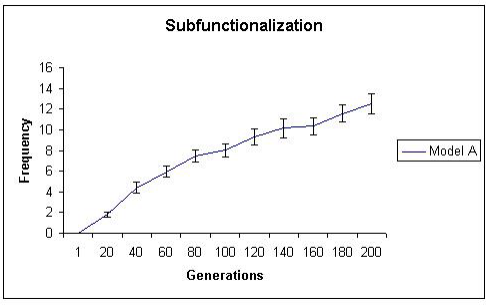
The frequency of subfunctionalization without neofunctionalization is shown for simulation A, where subfunctionalization is allowed. Subfunctionalization alone never represents a large fraction of cellular genomic fates.

**Figure 4:**
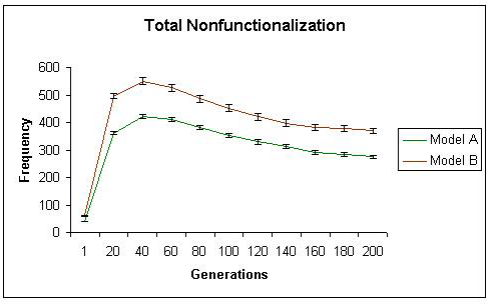
The total rate of nonfunctionalization is shown for simulation A in green and simulation B in red. Simulation B shows a much higher rate of nonfunctionalization at all evolutionary times.

**Figure 5:**
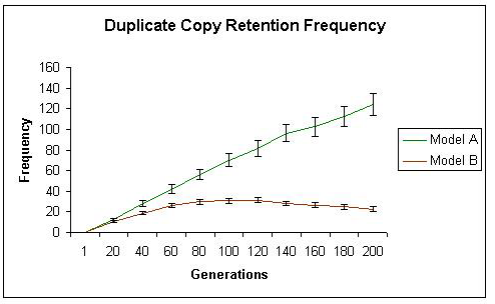
The total rate of duplicate copy retention without redundancy through either neofunctionalization or subfunctionalization is shown for simulation A in green and for simulation B in red. Allowing subfunctionalization to occur results in a different retention profile, with a much higher rate of duplicate copy retention.

**Figure 6:**
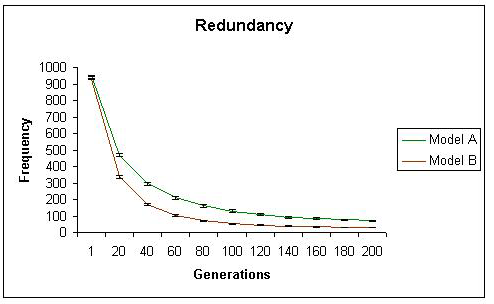
The frequency of redundant copies decreases with time in both simulation A (green) and simulation B (red). This occurs faster in simulation B, due to the faster rate of nonfunctionalization.

The role of subfunctionalization as a transition is based upon increasing the mutational space accessible to duplicates to neofunctionalize with removal of selective constraint at a binding site. This walk will differ for different lattices (and for different real proteins), modulating the importance of the effect of subfunctionalization. The rate of neofunctionalization in the absence of gene duplication (the emergence of new function in orthologs) is also related to the accessibility of this pleiotropic walk, but is expected to be even slower than that of neofunctionalization in the absence of subfunctionalization.

While it is not possible to systematically analyze duplicated fates and classify duplicated proteins as neofunctionalized, subfunctionalized, or redundant, this study has implications for our understanding of the role of duplication in the evolution of genomes. Protein segments that have lost function but are stably maintained in an expressed form will drift through sequence space until they achieve a function that makes them the targets of selection. Looking back to the origin of chordates, there is little doubt that gene duplication and the evolution of new function (as evidenced by annotation) went hand in hand. However, it may be that subfunctionalization initially played an important role in preserving copies that subsequently neofunctionalized over the past hundreds of millions of years.

## Conclusion

Subfunctionalization has previously been shown to increase the retention rate of duplicate genes using a similar approach [13]. However, when neofunctionalization is included as a possible fate for duplicate genes, subfunctionalization is still important in short time frames after duplication. However, with increasing time, subfunctionalization decreases in importance and its role seems to be to preserve duplicate copies for eventual neofunctionalization, a role as a transition state. Subfunctionalization can still play an important role with larger finite population sizes, but the importance of neofunctionalization as a terminal fate becomes even more dramatic with increasing population size.

## Methods

### Lattice model for protein sequences

We considered a simplified model of evolving proteins. Our model consisted of a chain of 64 random (codon derived) amino acid monomers on a three dimensional 4 × 4 × 4 cubic lattice, simulating a folded protein. Gene sequences were selected randomly and lattices folded as below. Two adjacent binding sites were randomly selected on each lattice. Three peptides were then designed: two that bound specifically at each site and a third that was bound by neither site, as shown by the binding energies in Table 1.

### Lattice folding and selection

Each amino acid was embedded at a single lattice point with distinct amino acids correspond to a distinct lattice point. All amino acids were considered to be of uniform size connected with covalent bonds of uniform lengths. A protein fold corresponded to a self avoiding walk over the embedding. The walking algorithm tracks the sites visited to avoid visiting them again. A contact was assumed to exist between two residues if they were not adjacently covalently connected but were on adjacent lattice points. The energy of the protein in a particular conformation was calculated according to the formula,



where *γ*(A_i_, A_j_) is the contact potential between residue type A_i _at position i and residue type A_j _at position j, and U_ij _is equal to one if residues i and j are not adjacent in sequence but are on adjacent lattice sites, and zero otherwise. The value of *γ*(A_i_, A_j_) is obtained from the symmetric interaction matrix given by Miyazawa & Jernigan [14].

### Evolution of lattice proteins

We have simulated two evolution models. Model A, corresponding to the evolution of a set of ten protein sequences (shown in Table 1) evolving to the alternative fates shown in Figure 1 and Model B corresponding to the evolution of the same set of proteins evolving without allowing subfunctionalization. Cells that did not bind ligands A and B (Model A) and ligands A and B in the same molecule (Model B) died. All molecules also needed to fold to be active.

In each model, we considered 1000 haploid cells that did not recombine between copies (independently for all ten gene duplicate pairs), with a protein molecule evolving according to a Poisson distribution with an average of 1 DNA mutation per gene per generation after the duplication event with a transition to transversion ratio of 2. After every generation, 10% of genes were knocked out at random to simulate mutations to transcriptional regulatory sequences and the cells were subsequently divided into the different fates shown in Figure 1. The next generation of cells was picked randomly from the living cells of the previous generation to keep a constant population size, with a 5% selective advantage to the neofunctionalized cells according to the Wright-Fisher selection model [15]. At the start of each generational round, each cell was viable with two gene copies to bind each set of ligands, as shown in Table 1.

## Authors' contributions

SR wrote all programs and carried out the simulations and analysis. DAL conceived of the study, supervised its execution, and wrote the manuscript.
